# Acquired immunity and asymptomatic reservoir impact on frontline and airport ebola outbreak syndromic surveillance and response

**DOI:** 10.1186/2049-9957-3-41

**Published:** 2014-10-29

**Authors:** Ernest Tambo, Zhou Xiao-Nong

**Affiliations:** Sydney Brenner Institute for Molecular Bioscience, School of Medical Sciences & School of Public Health, University of the Witwatersrand, Johannesburg, South Africa; Chinese Center for Disease Control and Prevention, National Institute of Parasitic Diseases, Shanghai, 200025 People’s Republic of China; WHO Collaborating Centre for Malaria, Schistosomiasis and Filariasis, Key Laboratory of Parasite and Vector Biology, Ministry of Health, Shanghai, 200025 People’s Republic of China; Département de Biochimie et Science Pharmaceutiques, Université des Montagnes, Bagangté, République du Cameroun

**Keywords:** Immunity, Asymptomatic, Reservoir, Syndromic, Surveillance, Ebola, Response

## Abstract

The number of surveillance networks for infectious disease diagnosis and response has been growing. In 2000, the World Health Organization (WHO) established the Global Outbreak Alert and Response Network, which has been endorsed by each of the 46 WHO African members since then. Yet, taming the dynamics and plague of the vicious Ebola virus disease (EVD) in African countries has been patchy and erratic due to inadequate surveillance and contact tracing, community defiance and resistance, a lack of detection and response systems, meager/weak knowledge and information on the disease, inadequacies in protective materials protocols, contact tracing nightmare and differing priorities at various levels of the public health system. Despite the widespread acceptance of syndromic surveillance (SS) systems, their ability to provide early warning alerts and notifications of outbreaks is still unverified. Information is often too limited for any outbreak, or emerging or otherwise unexpected disease, to be recognized at either the community or the national level. Indeed, little is known about the role and the interactions between the Ebola infection and exposure to other syndemics and the development of acquired immunity, asymptomatic reservoir, and Ebola seroconversion. Can lessons be learnt from smallpox, polio, and influenza immunity, and can immunization against these serve as a guide? In most endemic countries, community health centers and disease control and prevention at airports solely relies on passive routine immunization control and reactive syndromic response. The frontline and airport Ebola SS systems in West Africa have shown deficiencies in terms of responding with an alarming number of case fatalities, and suggest that more detailed insights into Ebola, and proactive actions, are needed. The quest for effective early indicators (EEE) in shifting the public and global health paradigm requires the development and implementation of a comprehensive and effective community or regional integrated pandemic preparedness and surveillance response systems tailored to local contexts. These systems must have mechanisms for early identification, rapid contact tracing and tracking, confirmation, and communication with the local population and the global community, and must endeavor to respond in a timely manner.

**Electronic supplementary material:**

The online version of this article (doi:10.1186/2049-9957-3-41) contains supplementary material, which is available to authorized users.

## Multilingual abstract

Please see Additional file 1 for translations of the abstract into the six official working languages of the United Nations.

## Background

The current widespread Ebola epidemic is estimated to infect 20,000 people before it is contained by early 2015. The total number of probable and confirmed cases of the Ebola virus disease (EVD) in the five affected countries as reported by the Ministries of Health of Guinea, Liberia, Nigeria, Senegal and Sierra Leone is 8914 cases and has claimed more than 4500 deaths so far. More than 40% of the total number of cases have occurred and are concentrated within a few localities. The average case fatality rate is 52%; this ranges from 42% in Sierra Leone to 66% in Guinea. A separate outbreak of the EVD, which is not related to the outbreak in West Africa, was laboratory confirmed on 26 August 2014 by the Democratic Republic of Congo (DRC). There have been 92 cases and 48 deaths so far [1]. Priority responses and actions are needed to tackle the ongoing Ebola crisis in West Africa and this requires improvements in access to diagnostic technologies and healthcare resources, as well as improved surveillance and communication. As it stands, there is little incentive for vulnerable communities to seek professional diagnosis of suspected Ebola. Most people with the flu and febrile illnesses self-medicate and are treated at home or by traditional healers/practitioners, making difficult to define the true extent and nature of the outbreak [2].

Syndromic surveillance (SS) has been advocated and used to monitor illness syndromes and events, and to detect epidemics and bioterrorist attacks early, thus increasing and ensuring that the response from public health departments is timelier. However, its effectiveness and usefulness in Ebola outbreak surveillance remains unclear [2, 3]. Up until now, no unified definition for SS coupled with limited predictive abilities of emerging diseases and Ebola seroconversion with no associated clinical signs has been determined. The source of high rate of health workers and volunteers infections and death is worrisome and urgently required further investigations, probably resulting from poor adherence and poor compliance to protective measures protocols and standard operating procedures, hard to implement standard clinical and laboratory operating procedures in such challenging environments, stress and anxiety poor incentives and lack of health insurance of those high risk health and humanitarian workers and quality assurance of the local and humanitarian protective products, the protocols. A number of remarkable similarities exist between the humoral responses to filoviruses, in particular the Ebola virus, and the response to HIV-1 infections, and these have been invaluable in demonstrating that antibodies can indeed provide protection against a virus [4]. Therefore, the efficiency of frontline and airport SS is compromised by a number of confounders/factors, such as acquired immunity, human-animal host asymptomatic reservoirs, inadequacies in diagnostic tools, infrastructure of health and social support systems, and various biosocial, environmental and climatic factors. These are still poorly understood in Ebola bottlenecks and thus hinder the establishment of adequate and reliable responses to prevent new cases, control further infections, and contain the ongoing geo-distribution trend and pattern in West and Central Africa.

It is common in SS that what is to be detected is unknown, and with asymptomatic and acquired immunity Ebola appears to present long incubation periods and prodromes. In order to test detection methods, a simple disease simulation model examination of a spectrum of ultraviolet biosensors of temperature (fever) sensitivity and specificity can provide advantages and limitations in frontline as well as Airport syndromic surveillance and could be applied at all times in monitoring any early symptoms in the absence of disease outbreak. It is then sufficient to run the detection method on a subset of simulated disease scenarios or on the challenges present in predicting unbiased diseases that are sufficiently different in order to determine the strengths and weaknesses of different methods at the point of care [4, 5]. Specific definitions for SS are lacking, and the name itself is imprecise. Certain programs monitor just surrogate data sources (e.g., over-the-counter prescription sales or school absenteeism) rather than specific disease syndromes. Meanwhile, certain well-defined disease or clinical syndromes (e.g., hemolytic uremic syndrome or Kawasaki’s syndrome) are not included in syndrome definitions, often leading to confusion about what “syndromic” surveillance actually monitors.

The different types of public health surveillance systems for early detection of outbreaks include:

1. Early warning systems in the region, disease control policies to restrict border crossings, as well as sales and accustomed consumption of bush meats, which have been ineffective implemented and sustained in diseases or outbreak surveillance and response. In addition, the handling of information by politicians, who have a history of partisan gain and constantly manipulate the media for political agendas, has been worsened over the years, underscored by corruption and nepotism. These coupled with weak health programs—continues to jeopardize genuine efforts to convey timely, trustworthy, and reliable information in a language accessible to the most vulnerable and remote communities [6, 7]. Symptom-based surveillance relies on self-reporting, health-based or routine admission procedures with clinical examinations, specific diagnosis, and recognition and reporting by clinicians to public health authorities/departments. Smart phones should play a greater role in these systems as well. Laboratories and other bodies in the region have shown that routine SS systems can be designed to rely on mobile phones which have become ubiquitous in West Africa. Some researchers forecast that mobile internet use in Africa will increase 20-fold in the next five years, and will consequently double the growth rate in rest of the world, and could be of potential use for individual or community-based surveillance.
2. Prodromal surveillance of flu or febrile like diseases has low specificity and may have insidious uncertainty based on differential diagnosis and overlapping early clinical multiple syndrome syndemics that trigger false alarms compared to post exposure, which can cause severe or life threatening disability. The type of prodrome varied from one individual to the next based on previous illness trends, pre-immunity, early indicators for response, over counter or self-medications, and various genetic, ecological and environmental factors [2, 6, 7]. In West Africa, the poor evidence-oriented approaches and tools, principles, and guidelines for communication before or during an outbreak require pivotal redesigning based on local contexts and taking into account social media and web-based health information and communication. The usefulness of WHO tools, guidelines, and recommended practices in Ebola outbreak prodrome surveillance in this context is underscored by local limitations, gaps in knowledge, and other challenges. Prodromal surveillance tools could be very timely and vital in the realm of emergency communication, advice, behavior change, hygiene and sanitation, and provide an integrated grassroots-based participatory approach. This could lead to more effective prevention, thus curbing the spread and containing the outbreak at the different levels of the disease, and at different places in time and space [3, 6].
3. Outbreak detection systems urgently require reliable, effective, and cost-effective tools and interventions which allow for constant access to diagnostic tools and personal protective equipment in healthcare centers across the region [7].
4. Information system-based sentinel surveillance.
5. Biosurveillance systems are used in practice to augment classical outbreak investigations. The major advantages of syndromic systems include sensitivity, timeliness, and flexibility, and being able to provide data for situational awareness. However, biosurveillance precincts lack in specificity, rely on chief complaint data, and lack formal training for users. Linking syndromic data to triage notes and medical chart data would substantially increase the value of biosurveillance in outbreak investigations and thus reduce the health burden [8, 9].
6. Laboratory-guided detection of disease outbreak surveillance systems relies on detection and monitoring of biothreats enabled by laboratory methods of diagnosis and identifies trends in biosurveillance research. It is based on three approaches, namely: (1) laboratory-initiated infectious disease notifications, (2) SS based on health indicators, and (3) genotyping-based surveillance of biothreats. The insufficient and delayed support for biosurveillance alerts for public health users and the inadequate integration of surveillance signals into action plans remain the major barriers, and require coordination between syndromic and laboratory-based surveillance for efficient public health outbreak monitoring and response [10].
7. Health indicator surveillance provides authorities with vital health indicators such birth rates, motility rates, and life expectancy. However, trustful and reliable information and communication, health education on preventable fatalities and cautious behavior are also required in order to prevent fear, panic and community resistance to stem out the spread of the disease outbreak.
8. Digital or electronic bio-epidemiology surveillance systems, including social media networking and web-based systems, provide valuable channels for timely collection of public health data; give information on the early detection of, and response to, disease outbreaks; and enhance situational awareness to communities. The creation of blogs and user-generated content has turned social networking into a conversation space in which everyone can participate. However in West Africa, the low level of literacy and high inequality indices compromised the usefulness of such tools to trace and map the Ebola outbreak, as compared to their usefulness and effectiveness in a community with high literacy, for example during the SARS and H9N7 influenza outbreak in Hong Kong and mainland China. Social media (e.g., Twitter, Facebook) and web-based communication provide epidemiological knowledge dissemination, and creates virtual communities based on shared values about critical outbreak perceptions, seriousness of the crisis, and population evidence-based guidance. However, it is not subject to experts or authorities’ advice and assessment, and doesn’t receive guidance from associated communication or information risk management and security. This requires further development and attention. The implications of social media and web-based information and communication in creating fear, anxiety and stigmatization about Ebola in some communities in West Africa are noteworthy. During this outbreak, web-based activities were also responsible for fuelling rumors that led to counter-productive behaviors. Improved communication between reliable health officials and the media, community leaders, health professionals, and the general public is necessary to reduce misinformation and improve compliance with Ebola prevention and control measures that have proven effective. These include population dynamics of emerging infections and the optimal design of monitoring and management strategies in prevention, control, and containment of Ebola in other countries in Africa [11].

Nonetheless, the term “syndromic surveillance” (SS) has persisted to describe this kind of surveillance as its fundamental goal is to identify signs and symptoms of illness clusters early before diagnoses are confirmed, report to public health authorities or agencies, and mobilize responses rapidly. Syndromic surveillance targets the threshold number of early symptomatic cases allowing outbreaks to be detected earlier than conventional reporting of confirmed cases would allow [2, 6, 7]. Response protocols for investigating SS alerts present some limitations in most endemic countries with syndemics acquired/partial immunity, diagnosis and identification of co-infections clusters, and sources of exact human-animal reservoirs [6]. Contact tracing and epidemiological case investigation of the nature and severity of the outbreak could provide timely and scientifically reliable information to curb the risk of propagation. Hence, more effective surveillance and response systems such as point-of-care Ebola molecular typing and immune-detection assays and rapid diagnostic kits in frontline and airport detection are urgently needed. For Ebola outbreaks, SS is able to provide the early symptom (prodrome) period before clinical or laboratory confirmation of the disease, as is explained below, although difficult in endemic areas in Africa where many tropical diseases with similar and/or differential signs and symptoms co-exist.

Ebola will continue to be a global threat if prompt and effective commitment is not directed towards control and containment. Surveillance and response systems are of interest in public health and veterinary epidemiology for the early detection of the emergence or re-emergence of infectious diseases. In relation to several confounders of Ebola outbreaks such as flu-like signs and symptoms of unknown sources, SS, which consists of the routine monitoring of indicators to detect adverse health events, may allow for early detection depending on continuous indicator measurements and sensitivity and specificity (timeliness tools for the detection or diagnosis of diseases outbreak emergence). The limitations in detection and spot diagnosis of asymptomatic reservoirs and pre-existing immune confounders have been the challenges since Ebola broke out Guinea in December 2013, was detected in March 2014, and finally spread to Liberia, Sierra Leone, and Nigeria. It is the most severe outbreak of Ebola since the discovery of the virus in 1976, with the number of cases from the current outbreak outnumbering the combined cases from all known previous outbreaks. The WHO has declared the Ebola outbreak in West Africa to be a Public Health Emergency of International Concern and called for action [12, 13]. Below, we outline the characteristics of the SS approach.

1. The concept and application of SS is doubly attractive because in addition to its potential to increase the speed and effectiveness of the public health response to natural or deliberate disease outbreaks with a certain degree of assurance, it costs far less to implement than traditional, labor-intensive approaches to disease surveillance (both should complement each other) [14]. However, the ability of SS to reduce disease-related morbidity and mortality remains to be demonstrated, as does its cost-effectiveness and warning devices. It will be critical to assess its utility, sensitivity, and accuracy in outbreak or bioterrorism within the context of health systems that respond to both “true” and “false” alarms in infectious disease syndemic settings. This involves the collection of information and clinical data that might indicate if an infectious disease outbreak might be happening in the community and whether it warrants further public health response. Before an outbreak occurs, little is documented in health centers and by airport active SS, except for passive checks of yellow card immunization for BCG, polio, and hepatitis vaccines programmes, in addition to medical referrals for passengers requiring medical or surgical interventions abroad. During the ongoing outbreak of Ebola in West Africa, SS, along with collaborative efforts between local health departments, has been used on patients, ground staff, health workers, passengers across community/national borders, and airports across Africa and in some other major airport hubs worldwide.
2. This approach is confronted by a lack of effective and accurate spot invasive frontline and airport rapid diagnostics tools, district and provincial health laboratories being equipped with little or no advanced molecular technologies, lack of drugs and vaccines to treat Ebola, inadequacy in coordinated Ebola frontline planning efforts in the community, as well inefficient or nonexistent community and national active infectious disease surveillance systems. Syndromic surveillance systems monitor existing descriptive data of these behaviors (e.g., school and work absenteeism, sales of over-the-counter medications, illness-related information, emergency room admissions for symptoms indicative of infectious diseases) for patterns or clusters of behaviors suggestive of an illness outbreak [7]. Hence, SS is not sufficiently equipped to control and contain Ebola in Africa due to its complex web of interactions and challenges.

The usefulness of laser thermal detection of febrile state or other characteristic symptoms of individuals in frontline and airport surveillance systems could be very challenging in hyper-, holo-, and meso-endemic settings, and present several limitations with the rampantly increasing confounders of poverty-related diseases in Africa and elsewhere. In addition, it should be noted that several factors such as travel syndrome, menopausal or post-menopausal syndrome in women and other lifestyle stressors associated with an increase in temperature—although normal—can trigger false alarms. Moreover, syndemics and partial acquired immunity in the region poses concerns about the spread and the burden of the disease due to asymptomatic reservoirs and the long latency period of infection [15, 16].

Efforts should be devoted to enhancing research and developing innovative, more sensitive detection and diagnostic tools for early-stage epidemic warning and preparedness in frontline and airport spot surveillance mechanisms and response, rather than increasing the use of empirical broad-spectrum detectors.

### The bottlenecks to Ebola outbreak frontline and airport syndromic surveillance and response systems

1. Screening and diagnostic tools

Diagnosis is “the cornerstone of effective outbreak and disease control and prevention efforts, including surveillance” [17]. Current challenges in diagnosis of Ebola by frontline and airport surveillance systems underscored the existing detection and diagnostic tools, and highlighted the importance of combining diagnostic needs with appropriate technologies. The need for rapid, accurate, inexpensive, and robust diagnostic low-detection thresholds can be met by recent advances in genomics, proteomics, and material science; profitable public-private partnerships; and sustainable profits in low-resource settings. The continued development and deployment of efficient, low-cost diagnostic platforms is essential for containment. Detection methods suitable to local/international standard laboratories or sentinel for imported cases epidemiology must be validated prior to transition from malaria sustained prevention and control programs and interventions. The importance of developing and implementing sensitive diagnostic approaches to accurately quantify and monitor Ebola reservoirs is imperative in curbing the persistent transmission dynamics, and preventing, controlling, and containing the disease given Africa’s engagement in achieving the Millennium Development Goals (MDGs), and in the London Declaration International Health Regulations (2005), the Universal Human Rights Declaration, and the New Partnership for African’s Development (NEPAD) in Africa. However, a number of challenges remain to be overcome before deployment of rapid, low-cost, sensitive, and specific point-of-care disease diagnostics become a reality.

- ● ***Spot frontline and airport surveillance using laser imaging of febrile conditions*** with a latency period of 2–21 days for clinical manifestations require rethinking, more research, and funding for the development of simple, rapid, field adaptable, and effective detection tools in asymptomatic, presymptomatic, and symptomatic cases, to be used in addition to spot airport passengers’ diagnostic kit(s).
- ● ***Immune variability across African countries with*** syndemics is poorly understood, although it is believed that populations develop varied degrees of acquired/partial to complete immunity resulting from repeated exposure to infectious diseases, and can carry a certain load of virus for months or years before it becomes a clinical manifestation of the Ebola disease [4, 18]. The concept of immunization or vaccination as described by Edward Jenner (1749–1823) observed that people with cowpox infection developed immunity to smallpox, with several lifelong survivors. Hence, smallpox was declared eradicated in the wild in 1980 after a worldwide immunization campaign took place similar to the ongoing polio eradication with effective immunity response to outbreak depending on the age and level of individual antibodies (cell-mediated immunity) and protective threshold. Consequently, large asymptomatic population-animal reservoirs in the case of the Ebola infection in Africa may not be surprising, and further screening of Ebola exposed and non-exposed populations is required. What are the lessons and challenges learned in shaping future research priorities? This makes the need for in-depth knowledge on the exposure of viral infectious (e.g., measles, yellow fever, chickenpox, or HIV/AIDS) even more urgent as this can contribute to Ebola resurgence and the quest for an Ebola vaccine [4].
- ● ***Genetic and clinical variability have shown that genetic make-up*** or traits vary from one ethnic group to another, and within and between populations with different clinical manifestations, but the relationship between population genetic changes and Ebola seroconversion and progression over time and space is still poorly understood. The precise role and efficacy of biosurveillance in public health has yet to be determined, as well as the limitations of SS systems to detect Ebola infection or other outbreaks. Health professionals should continuously aspire to accurately diagnose and treat patients, as well as to identify public health outbreaks or emergencies, combined with adequate local integration of infrastructure, facilities, and capacity building.
- ● ***Environment, ecological, and animal interface, and encroachment factors*** due to landscape use and misuse, mining, deforestation, forest degradation, wildfire, conflicts/wars, and man and animal interactions also require further research.

2. Infrastructure and capacity of health and social systems

Challenges in Ebola control and containment in West Africa are obvious due to a lack of humanitarian response models for fragile and under resourced health systems, and the local government and affected community’s inability to contain the wide spread of the disease. These challenges include: insufficient regional and international political commitment, insufficient resources and funding, lack of an Ebola vaccine or drug, detection and diagnostic limitations, and a lack of resources or infrastructure to support such activities. Additional challenges include inadequacies in programs and approaches, weak or nonexistent primary healthcare infrastructure, poor access to health facilities, and a lack of effective mental, traumatic or neurological assessment tools, as well as national and regional functioning early-warning alert and surveillance response systems. Other associated factors include social media and web-based information and communication; an artificial country colonial landscape demarcation and barriers amongst African countries with cross-border families; marriage; employment and commercial/trading activities; sociocultural realities and practices; attitudes to care seeking and utilization; environmental and ecological risk factors; human-animal migration and movement dynamics; conflicts/wars and violence in the region; intense mining activities in the region with an associated impact on the political sphere; and the socioeconomic, ecological, and epidemiologic impact of Ebola and others infectious and chronic diseases.

3. Acquired or partial immunity of local populations

Due to the scarcity of data on immune parameters and exposure doses, the exact impact of the disease on humans is hard to quantify. Evaluating ‘acquired immunity’ may improve outbreak estimates when evaluating the risk of microbial illness from food or environmental exposures. This suggests that some current approaches may significantly overestimate their role in causing such illnesses. Immune status is a major factor in susceptibility to disease outbreak, and the impact of acquired immunity to a pathogen needs careful insight when assessing the potential health risks of outbreaks and other infectious diseases of different sources of exposure, including (1) low-frequency, low-dose exposure (recreational water); (2) low-frequency, high-dose exposure (consumption of raw chicken liver); (3) high-frequency, low-dose exposure (direct contact with sheep and goats, i.e., farmers); and (4) high-frequency, high-dose exposure (visiting petting zoos, wildlife hunters or bush meat sellers/consumers). The public health community should also take acquired immunity into account in order to improve estimates of the potential impacts of infectious diseases and to assist in preventing and managing outbreaks. Further studies to better characterize and quantify the effects of acquired immunity on Ebola outbreaks are also needed. In humans, there may be apparent ecological, ethnic susceptibility and geographical landscape variations, but it is always important to disentangle such factors—as well as climate, nutrition, environmental, and economics drivers—from those that might be genetically determined in both animal and human transmission dynamics.

4. Reservoirs of Ebola virus carriers in both humans and animals

Ongoing efforts to control and contain the Ebola outbreak have been limited by the estimated mass asymptomatic population and animal-human reservoirs, which enhance the tenacious transmission dynamics between and within some communities, provinces, and across African countries and elsewhere. Viral infectious diseases such as HIV/AIDS, hepatitis coupled with malaria, tuberculosis, and other neglected and emerging infectious diseases are rampant in Africa [6]. Assessment of transmissibility requires tools that can accurately identify the various developmental stages of the animal-human and/or human-human interphases. Moreover, in most epidemic areas, asymptomatic carriers are not uncommon and, as potential carriers, represent a significant reservoir for Ebola transmission regardless of successful local interventions. These are the challenges to the current humanitarian and national prevention, control, and containment programs [19]. Many of these asymptomatic infections may be present at densities below the limit for microscopic and rapid diagnostic tests threshold detection and thus lead to underestimation persistence of the epidemic burden and probably resurgence.

There is very limited, if no, accurate information or data available on submicromolar asymptomatic carriers or presymptomatic surveillance, the detection and diagnosis responsible for Ebola virus survival, and persistent transmission on susceptible populations. Paucity information pertaining to the current status of the effectiveness of microscopic and rapid diagnostic test tools necessary for Ebola control and containment interventions, except for molecular confirmation of cases done in very few selected research centers in Africa and across the country since the first Ebola outbreak in 1976 is also lacking.

The development and deployment of active surveillance at all levels coupled with monitoring and evaluation (M&E) of outbreak risk factors and transmission dynamics in early active detection asymptomatic and presymptomatic cases, as well as prompt management of either local or imported cases, is paramount to understanding the viral seroconversion dynamics in suspected communities and travellers in Africa and worldwide. Sensitive and effective serological, immunological, and biochemical Ebola biomarkers that can be used in these remote communities with uncertain or low animal and population reservoirs alongside spot airport testing, mass deployment in mapping geographical distribution, evidence informed policy decision, and prompt interventions—are also essential. Understanding the Ebola epidemiological trend and patterns including reservoirs and transmission dynamics can provide valuable information for the success of Ebola control and containment strategies.

### Recommendations

Although SS response systems are able to detect Ebola outbreaks earlier than traditional surveillance, it will be more efficient for these systems to prepare for the standard operational protocol to avoid unexpected occurrences of events. Therefore, we recommended the strengthening of the following activities in order to improve the frontline and airport SS responses to Ebola outbreaks:

1. **Improving case investigation, tracking of susceptible populations, and quarantine period**
  Most African countries are challenged by insufficient or nonexistent facilities, a lack of qualified personnel, and the vicious cycle of poverty. Cultural practices and myths, challenges in African traditional and alternative medicine implementation in healthcare systems, and attitudes towards health seeking should also be noted. The poor landscape mapping, rural and urban town planning, and especially the poor or nonexistent accessible roads to these communities are other contributing factors. Tracking can be very difficult in areas with poor documentation habits, lack of appropriate reporting or a contact tracing system, uncontrolled migration and population movement across borders, unlimited cross-border marriages and trade, as well as animal in- and out-flow of foreigners at the entrance or departure terminuses (airport) in Africa and elsewhere. The porous nature of West African country borders stresses the need for automated robust, high-sense human and animal health and movement detectors, in partnership with communities and governments so appropriate data can be collected to answer essential Ebola questions. Public health surveillance and powerful analytical tools are needed to accurately interpret the findings.
  The strengthening of the epidemiological capacity through surveillance response systems at the local level needs to be advocated in order to inform the interpretation of syndromic findings in light of “local epidemiological peculiarities,” as well as to ensure a rapid response to syndromic alerts. Under enabling conditions, community-based mobilization and empowerment in recognizing, informing and active case investigation and contact tracing could build strong relationships between public health and healthcare providers in effective early alert, prevention, control of current and future outbreaks. These relationships are critical for reliable and effective Emergency outbreak response and follow up epidemiological investigation, and for evidence policy-building regardless of the type of intervention [20, 21].
2. **Nurturing “One Health and One world” surveillance and response systems**
  Infectious diseases primarily affecting animals can have direct and indirect impacts on humans including significant economic consequences. Two important factors can contribute to the proliferation of zoonotic diseases: the explosive growth of human and domestic animal populations, and the increasingly close physical proximity within which humans and domestic and wild animals live [6, 20]. Timely identification of current and future emerging microbial threats (on the order of SARS, the West Nile virus, and H5N1 avian influenza) will require an integrated international approach to disease surveillance. However, progress has been hampered by a variety of mining, environmental, climatic, socioeconomic, and political factors, in addition to a weak and fragile or nonexistent surveillance infrastructure and technology, and inadequate expertise in Africa.
  Success in Ebola control and containment requires a comprehensive and integrated strategy in human disease surveillance among the underserved populations that live in close contact with bat fruits, gorillas, and other wildlife animals. This strategy should incorporate capacity building, training, and empowerment of the local community by integrating simple data collection with basic laboratory diagnosis to identify the link between human outbreaks of the Ebola virus, and poaching, the consumption of bush meats, basic hygiene measures such as hand washing and cooking meat thoroughly, overall food safety in communities, and early warning of outbreaks in animals [20].
3. **Fostering integrated active surveillance response systems**
  In view of the recognized potential of SS systems, there are many practical concerns about sensitivity and false-positive rate trade-offs and the time required to accumulate enough evidence of an outbreak to trigger a detection algorithm, as well as on the available control strategies of the local, national, and regional public health practice and utilization of these systems. The broad and multifaceted practices of surveillance approaches are used to monitor the progress and outcome of interventions to mitigate or stop the progression of an outbreak, including of economically and ecologically important animal or plant species and the transmission of zoonotic diseases among animal and human populations over space and time, as well as to predict future transmission patterns [20, 22]. Currently, disease outbreak surveillance and detection relies heavily on the astute individual: the clinician, the veterinarian, the grower, and the livestock manager noticing both routine and suspicious symptoms and bringing them to the attention of the public health or veterinary community including academics and zoological parks. Most developed countries have a surveillance system in place and the ability to detect and diagnose human and animal diseases. Innovations and strategies for the surveillance and detection of human and animal diseases, and assessing the resource needs and opportunities for improving and coordinating infectious disease surveillance, early detection, tracing, case investigation, prompt reporting, and management are needed in upholding health systems strengthening and future sustainable development in most under resourced countries [21, 22].
  Technological advances in disease surveillance and detection that have benefited public health surveillance such as rapid, automated, and sensitive biosensors; portable sampling and assay systems; and DNA-based diagnostic tools remain to be adapted to track animal diseases. Models and interventions incorporating M&E systems and true coordination and collaboration would enable optimal surveillance response, thereby driving policy and action, with a feedback process to facilitate continuous evolution and adaptation [20, 21]. Information would be drawn from a broad range of disciplines relevant to physical and mental health, as well as domestic and wild animal health and plant health, through the complementary processes of agent or disease surveillance and host and environmental monitoring with potential economic benefits of surveillance systems for all. Nevertheless, the release of surveillance information should be evaluated on a case-by-case basis, as trust is not built by merely sharing data, but by helping people understand information that is context-specific. Active engagement to discuss the perception of risk of outbreak and identifying priorities for action is also essential. Community or regional active surveillance systems, new effective rapid diagnostic methods, and prompt reporting have the potential to advance infectious disease control and prevention efforts in Africa and elsewhere. Although, the Electronic Surveillance System for the Early Notification of Community-Based Epidemics (ESSENCE), operated by the Department of Defense, allows epidemiologists to track—in real-time—syndromes reported in daily data feeds from regional hospitals and clinics, it is yet to be actively implemented in most Africa countries.
  Due to the persistent outbreaks of infectious diseases across Africa, it is crucial to analyze the applicability of surveillance response systems in order to improve the ability of hospital/health center triage systems to identify and appropriately treat patients who show symptoms associated with an emerging infectious disease, a threat of an infectious disease (e.g., influenza, SARS, and Ebola, as well as potential bioterrorism agents such as anthrax and smallpox), or an emerging infectious disease. Laboratory diagnosis may be possible by building a network of information about early warning alert and response systems that travels up or down the public health hierarchy, from the local to the international level and vice versa [20–22].
4. **Shifting towards an effective public and global health paradigm**

Due to globalization, health for all under the “One Health” initiate calls for immediate Ebola and other outbreak actions plans. A future, in which outbreaks and bioterrorism agents are continually reengineered to evade standard detection and diagnostic methods, as well as therapeutics, is imagined. Hence, Africa and the global community has no choice but to move from postsymptomatic to presymptomatic detection and diagnosis, and to prompt effective surveillance response systems that seek to benefit the global community. Ultimately, to reach the best-case scenario stage in which microbes are ubiquitous, constantly evolving, and adapting requires community and national surveillance policies to inform and guide action on the basis of importance, not for reaction and emergency to dictate priority [21, 22].

There is no magic bullet for changing paradigms; steady progress, albeit being slow, can be made through small successes. This needs to be properly recognized as an effective engine for change to educate the next generation of leaders early in their careers and encourage greater global, inter-, and trans-disciplinary awareness in future public health professionals.

The quest for EEE in shifting the public and global health paradigm to achieve the MDGs post the 2015–2030 agenda, the “One Health, One World” and other global health initiates requires:

- ● Community outreach and advocacy, and local and international mobilization to combat outbreaks in Africa and globally.
- ● Multidisciplinary approach studies to understand the drivers, determinant dynamics, and risk factors of persistent Ebola outbreaks.
- ● Strengthening south-south and public-private partnerships to build local capacity, health education, and empowerment in health and environmental community health for sustainable development.
- ● More research in host-based early-warning alert models and understanding of the contribution of context, culture, and ecosystems on asymptomatic/presymptomatic factors in Ebola pre-exposure diagnosis prior to the appearance of symptoms. Monitoring a person’s blood serum chemistry for changes that suggest a compromised health status or non-invasive sampling of breath and saliva is attractive in theory.
- ● Rapid molecular markers for mass population screening and diagnosis-based triage and increasing the effectiveness of quarantine or other social distancing measures including the development of synthetic antibody techniques for monitoring infection-related changes in protein levels.
- ● Monitoring the biological signatures of infectious disease devices: easily accessible (e.g., in the home), robust, inexpensive, and capable of quickly measuring thousands of Ebola outbreak spatiotemporal minimum effective data for mining variables in understanding the progression from asymptomatic to clinical Ebola cases and forecasting future Ebola trends and geo-distribution.
- ● More infrastructure and facilities in rural and remote areas, especially in mining African countries, as well as research and development (R&D) funding for Ebola drug and vaccine development.
- ● Development and implementation of country and cross/regional active and integrated community-based surveillance response systems and M&E initiatives to formulate alternative and innovate community/national recovery and rehabilitation programs, measures, and interventions post-Ebola outbreak surveillance and response systems.

## Conclusion

Given the considerable interdependence of surveillance, detection, and diagnostic activities and infectious diseases, it is not surprising that the key challenges identified in this paper can be overcome by innovative surveillance strategies and future prospects as described above. Early detection is essential to control and contain the spread of the Ebola outbreak. A disease such as this—in a profoundly interconnected world—requires active vigilance for rapid recognition, and prompt diagnosis, case investigation, and tracking of its causes and sources, as well as the mitigation of reliable and robust strategies and resources for an appropriate and efficient response. This paper illuminates the major gaps in frontline and airport Ebola control and containment, and provides structured opportunities for leaders, governments, academia, industry, and stakeholders to more robustly mobilize and combine resources. We examine issues of shared concern regarding research, prevention, detection, and management of the Ebola outbreak and other emerging and re-emerging infectious diseases.

## Electronic supplementary material

Additional file 1: **Multilingual abstracts in the six official working languages of the United Nations.** (PDF 272 KB)

## Abbreviations

EEE: Effective early indicators
EVD: Ebola virus disease
HIV/AIDS: Human immunodeficiency virus infection and acquired immune deficiency syndrome
MDGs: Millennium development goals
M&E: Monitoring and evaluations
R&D: Research and development
SARS: Severe acute respiratory syndrome
SS: Syndromic surveillance
WHO: World Health Organization.
