# Erratum: Acquired immunity and asymptomatic reservoir impact on frontline and airport Ebola outbreak syndromic surveillance and response

**DOI:** 10.1186/s40249-015-0042-4

**Published:** 2015-04-10

**Authors:** Ernest Tambo, Xiao-Nong Zhou

**Affiliations:** Sydney Brenner Institute for Molecular Bioscience, School of Medical Sciences & School of Public Health, University of the Witwatersrand, Johannesburg, South Africa; National Institute of Parasitic Diseases, Chinese Center for Disease Control and Prevention, Shanghai, 200025 People’s Republic of China; WHO Collaborating Centre for Malaria, Schistosomiasis and Filariasis, Key Laboratory of Parasite and Vector Biology, Ministry of Health, Shanghai, 200025 People’s Republic of China; Département de Biochimie et Science Pharmaceutiques, Université des Montagnes, Bagangté, République du Cameroun

## Erratum

After publication of this work [1], we noted an inversion of the first and family names of the author Xiao-Nong Zhou. The author list should be corrected as follows.

Ernest Tambo and Xiao-Nong Zhou.
