# Supplementary material for: Acquired immunity and asymptomatic reservoir impact on frontline and airport ebola outbreak syndromic surveillance and response
Source: Infect Dis Poverty. 2014 Oct 29;3:41. doi: 10.1186/2049-9957-3-41 (PMC4333876; doi:10.1186/2049-9957-3-41)
Supplement: Supplementary file 1 — Additional file 1: Multilingual abstracts in the six official working languages of the United Nations. (PDF 272 KB) [file 40249_2014_93_MOESM1_ESM.pdf]

Translation of the abstract into the six official working languages of the United Nations

تأثير المناعة المكتسبة ومُسْتَودَعُ الفيروس غير المصحوب بأعراض في الخطوط الأمامية والمطارات على المراقبة السريرية و الاستجابة السريعة لفيروس إيبولا

إرنست تامبو و زو زايو نونج

ملخص

تزايد عدد شبكات مراقبة الأمراض المعدية و شبكات الاستجابة في الفترة الأخيرة ، و في عام 2000 أسست منظمة الصحة العالمية شبكة تحذير واستجابة عالمية لتفشي الأمراض والتي تبناها ودعمها ال46 عضوا في منظمة الصحة العالمية منذ ذلك الوقت.

مع ذلك فإن عملية تحجيم ديناميكية واكتساح فيروس إيبولا المتوحش في البلدان الأفريقية هي عملية شاردة وممزقة نظرا لنقص المراقبة السريرية وعدم القدرة على اقْتِفاء المُلاَمِسين، و تحدي المجتمع ومقاومته لها، بالإضافة إلى نقص شبكات الرصد والاستجابة للأمراض، ضآلة /قلة حجم العلم والمعرفة عن طبيعة المرض، واختلاف الأولويات على كافة المستويات لأنظمة الصحة العامة لهذه البلدان.

بالرغم من القبول العريض و الواسع الانتشار لأنظمة المراقبة السريرية فلم يتم التأكد من قدرتها على تقديم إنذار مبكر وتنبيهات قبل تفشي المرض. وغالبا ما تكون المعلومات محدودة عن أي تفشي أو ظهور لأي مرض حتى و إن لم يكن متوقعا للتعريف به على المستوى المجتمعي أو القومي.

بالطبع، لا يوجد معلومات كثيرة عن الدور والتفاعلات بين العدوى بفيروس إيبولا والتعرض لمجموعة أوبئة الأخرى وتطوير مناعة مكتسبة، مستودع الفيروس غير المصحوب بأعراض، و التحويل المصلي (انْقِلاَبٌ تَفاعُلِيَّةِ المَصْل السيرولوجيّ) لفيروس الإيبولا

هل يمكن الاستفادة من دروس التطعيم ضد الجُدَرِيّ ، شلل الأطفال والإنفلونزا، و هل يمكن أن يعمل التطعيم ضد هذه الأمراض الخطيرة كتوجيه إرشادي؟ في أغلب البلدان التي يتفشى فيها الأوبئة تعتمد مراكز الصحة المجتمعية ومكافحة الأمراض ومنع دخولها بالمطارات على الوقاية بالتطعيم الروتيني السلبي و الاستجابة التفاعلية المتلازمية.

كان هناك أوجه قصور كثيرة في أنظمة المراقبة السريرية لفيروس الإيبولا في الخطوط الأمامية و المطارات في غرب أفريقيا من ناحية الاستجابة لعدد من حالات الوفيات الذي ينذر بوقوع مخاطر ويُقترح وجود حاجة للنظر بتمعن في مخاطر فيروس إيبولا واتخاذ إجراءات لاحقة . يتطلب البحث عن مؤشرات مبكرة فعالة لتغيير النموذج الصحي العام والعالمي تطوير وتنفيذ أنظمة المراقبة السريرية الشاملة و العالية في الكفاءة الاستعدادية المجتمعية أو الإقليمية تتناسب مع السياقات المحلية.

يجب أن تكون هناك آليات للأنظمة للرصد المبكر ، والتتبع السريع، والتأكد، والتواصل مع السكان المحليين و المجتمع الدولي ويجب عليها محاولة الاستجابة في الحال.

Translated from English version into Arabic by einass, through

TRANSLATORS
WITHOUT BORDERS

获得性免疫与无症状带毒者对埃博拉暴发后海关、机场的症状监测与响应的影响

Ernest Tambo and Zhou Xiao-Nong

摘要

从事传染病诊断与响应的监测网络数量正在增加。2000年，世界卫生组织建立了全球暴发预警与响应网络，那时46个世界卫生组织非洲成员国参与了此网络。但是，非洲国家由于不适当的监测和追踪措施、社区的阻止与抵抗、缺乏检测与响应系统、粗糙或溥弱的疾病知识与信息传播、以及不同公共卫生级别的优先措施不同等因素，使控制埃博拉传播与流行的力度分散与乏力。尽管症状监测系统已广泛接受，但是对暴发的早期预警和预报能力仍未有验证。有关暴发、新发或非预期的疾病被社区或国家认可的信息仍较少。确

实，对埃博拉病毒感染与暴露于其它多种疾病间、以及与获得性免疫、无症状带毒者和埃博拉血清阳转等间的关系与作用仍不清楚。是否可以从天花、小儿麻痹症和流感免疫与抗感染免疫中获得经验？在大多数流行国家，社区卫生中心和机场疾病预防控制站仅可以被动常规免疫和反应性症状处置开展工作。西非海关和机场的埃博拉症状监测系统在应对病例死亡数量的警示已显得很薄弱，提示亟需深入了解埃博拉并采取强化措施。有效地改变公共卫生和全球健康的早期指标有待于更好地实施或完善因地制宜的社区或区域性的综合全球储备与监测响应系统。该系统必须成为一个早期认别、快速跟踪、正确确认、与当地人群和全球行业保持沟通的有效系统，并且必须努力做到实时响应。

Translated from English version into Chinese by Zhou Xiao-Nong

Incidence de l'immunité acquise et des réservoirs asymptomatiques sur la surveillance syndromique et l'intervention en cas d'éclosion d'Ebola en première ligne et dans les aéroports

Ernest Tambo et Zhou Xiao-Nong

Résumé

Le nombre de réseaux de surveillance des diagnostics et des interventions liés aux maladies infectieuses connaît une augmentation. En 2000, l'Organisation mondiale de la Santé (OMS) a mis en place le Réseau mondial d'alerte et d'action en cas d'épidémie (GOARN) qui a été approuvé depuis lors par les 46 pays africains membres de l'OMS. Pourtant, le contrôle de la dynamique et de la vague de la maladie vicieuse à virus Ebola dans les pays africains s'est avéré sporadique et irrégulier en raison de la surveillance et d'une recherche de contacts inadéquates; de la méfiance et de la réticence de la communauté; d'un manque de systèmes de détection et d'intervention; de connaissances et d'information moindres ou limitées sur la maladie; et de priorités divergentes à différents niveaux du système de santé publique. En dépit d'une acceptation généralisée des systèmes de surveillance syndromique, la capacité de ces derniers à fournir des alertes et des avis précoces en matières d'éclosion n'a pas encore été vérifiée. Les renseignements sont souvent trop limités en cas d'éclosion, de maladie émergente ou de maladie inattendue, pour être reconnus au niveau communautaire ou national. En effet, nous en savons peu sur le rôle et les interactions entre l'infection au virus Ebola et l'exposition à d'autres syndémiques et le développement d'une immunité acquise, d'un réservoir asymptomatique et d'une séroconversion du virus Ebola. Des leçons peuvent elles être tirées de l'immunisation contre la varicelle, la poliomyélite et la grippe? Et l'immunisation contre ces maladies peut-elle servir de guide? Dans la plupart de pays endémiques, les centres de santé communautaires ainsi que le contrôle et la prévention des maladies dans les aéroports ne reposent que sur un contrôle de routine des immunisations passif et sur une intervention syndromique active. Les systèmes de surveillance syndromique du virus Ebola en première ligne et dans les aéroports en Afrique de l'Ouest ont présenté des lacunes en terme d'intervention, avec un nombre alarmant de décès, et suggèrent qu'une meilleure compréhension du virus Ebola et des mesures proactives sont nécessaires. La recherche d'indicateurs précoces efficaces en changeant le paradigme de la santé publique et mondiale nécessite l'élaboration et la mise en oeuvre de systèmes communautaires ou régionaux de surveillance, d'intervention et de préparation aux pandémies intégrés et adaptés au contexte local. Ces systèmes doivent disposer de mécanismes d'identification précoce, de suivi rapide, de confirmation et de communication auprès de la population locale et la communauté mondiale, et doivent permettre une intervention en temps opportuns.

Translated from English version into French by BM1983, through

Влияние приобретенного иммунитета и бессимптомного резервуара инфекции на синдромный эпиднадзор и ответные меры при вспышке вируса Эбола в аэропортах и приграничных территориях

Эрнест Тамбо (Ernest Tambo) и Чжоу Сяо-Нонг (Zhou Xiao-Nong)

Аннотация

Увеличивается количество сетевых организаций для диагностики инфекционных заболеваний и ответных мер. В 2000 г. Всемирная организация здравоохранения (ВОЗ) учредила Глобальную сеть предупреждения о вспышках заболеваний и ответных мерах, которая с тех пор была одобрена каждой из 46 африканских стран, членов ВОЗ. Тем не менее, снижение динамики и вспышки патологической геморрагической лихорадки Эбола (ГЛЭ) в африканских странах было неоднородным и неустойчивым в связи с недостаточным эпиднадзором и прослеживанием контактов, пренебрежением и сопротивлением сообщества, отсутствием систем выявления заболевания и ответных мер, скудных, слабых знаний и сведений о заболевании, а также отличающихся приоритетах на различных уровнях системы здравоохранения. Несмотря на широко распространенное признание систем синдромного эпиднадзора (СЭ), их способность обеспечивать ранние предупреждения и оповещения о вспышках заболевания все еще не подтверждена. Информация является часто слишком ограниченной при любой вспышке, возникновении заболевания или другой неожиданной болезни для того, чтобы быть распознанной как на общественном, так и на государственном уровне. Действительно, немного известно о роли и взаимосвязи между инфекцией геморрагической лихорадки Эбола и воздействием других синдемий, а также развитием приобретённого иммунитета, бессимптомного резервуара и сероконверсии вируса Эбола. Можно ли извлечь уроки из иммунитета к оспе, полиомиелиту и гриппу, и может ли иммунизация против этих заболеваний служить в качестве руководства? В большинстве эндемичных стран общественные центры здравоохранения, а также службы санитарно-эпидемиологического надзора и профилактики заболеваний в аэропортах полагаются исключительно на пассивную стандартную иммунизацию и реактивное синдромное реагирование. Системы СЭ вируса Эбола в аэропортах и приграничных территориях Западной Африки продемонстрировали недостатки относительно реагирования с тревожным количеством фатальных случаев, и предполагается, что необходимы более детальные исследования ГЛЭ и предупредительные действия. Поиск эффективных ранних показателей (ЭРП) в сдвиге общественной и мировой парадигме здравоохранения требует разработки и реализации всеобъемлющих и эффективных систем органов или регионально интегрированных систем подготовки к пандемиям, эпиднадзора и реагирования. Эти системы должны иметь механизмы для раннего выявления, быстрого отслеживания, подтверждения и передачи информации местному населению и мировому сообществу, а также должны стремиться реагировать своевременно.

Translated from English version into Russian by Alexander Vareiko, through

Impacto de la inmunidad adquirida y del reservorio asintomático sobre la vigilancia sindrómica y respuesta por brotes epidémicos de ébola en la primera línea y en aeropuertos

Ernest Tambo y Zhou Xiao-Nong

Resumen

Ha aumentado la cantidad de redes de vigilancia para la respuesta y el diagnóstico de enfermedades infecciosas. En 2000, la Organización Mundial de la Salud (OMS) estableció la Red Mundial de Alerta y Respuesta, que ha sido aprobada por cada uno de los 46 miembros africanos de la OMS desde ese entonces. Aún así, la domesticación de la dinámica y la plaga de la despiadada enfermedad del virus del ébola (EVE) en países africanos ha sido dispersa y errática debido a la vigilancia irregular y el seguimiento de contactos, la resistencia comunitaria, una carencia de sistemas de detección y respuesta, el conocimiento e información escasos/débiles sobre la enfermedad, y las prioridades discrepantes en varios niveles del sistema de salud pública. A pesar de la aceptación difundida de los sistemas sindrómicos de vigilancia (SS), su capacidad para proporcionar alertas tempranas de advertencia y notificaciones de brotes epidémicos aún no se ha verificado. Algunas veces la información es demasiado limitada para cualquier brote epidémico, o enfermedad emergente de otra manera inesperada para ser reconocida, sea en el ámbito de la comunidad o la nación. Por cierto, poco se sabe del papel y de las interacciones entre la infección por ébola y la exposición a otras sindémicas y al desarrollo de inmunidad adquirida, reservorio asintomático y la seroconversión del ébola. ¿Se pueden aprender lecciones de la inmunidad a la viruela, a la polio y a la gripe, y puede la inmunización contra estas enfermedades servir como guía? En la mayoría de los países endémicos, los centros comunitarios de salud y de control y prevención de enfermedades en aeropuertos únicamente se apoya en el control de inmunizaciones de rutina pasiva y respuesta sindrómica reactiva. Los sistemas SS de ébola en la primera línea y aeropuertos en África Occidental han mostrado deficiencias en virtud de responder con una cantidad alarmante de fatalidades en los casos, y sugieren que son necesarias más visiones detalladas acerca del ébola y acciones proactivas. La búsqueda de indicadores tempranos efectivos (EEE, por sus siglas en inglés) en cambiar el paradigma sanitario público y mundial requiere el desarrollo y la implementación de una comunidad integral y efectiva o preparación regional de pandemia integrada y sistemas de respuesta de vigilancia adaptados a contextos locales. Estos sistemas deben contar con mecanismos de identificación temprana, seguimiento rápido, confirmación y comunicación con la comunidad global, y deben esforzarse en responder en manera oportuna.

Translated from English version into Spanish by two2tango, through
